# Glandular odontogenic cyst: Comprehensive clinicopathological and immunohistochemical characterization

**DOI:** 10.4317/jced.62150

**Published:** 2025-01-01

**Authors:** Nicole Lonni, Breno Barbosa, Liane Souza, Daniella Couto–Vieira, Elena Rivero, Rogério Gondak, Ricardo Luiz Albuquerque-Júnior

**Affiliations:** 1 DDS, PhD Student. Postgraduate Program in Dentistry, Federal University of Santa Catarina, Florianópolis, Santa Catarina, Brazil; 2 DDS, PhD Student. Postgraduate Program in Dentistry, Federal University of Sergipe, São Cristovão, Sergipe, Brazil; 3 DDS, PhD. Department of Dentistry, Health Sciences Center, Federal University of Sergipe, São Cristovão, Sergipe, Brazil; 4 MD, PhD. Department of Pathology, Health Sciences Center, Federal University of Santa Catarina, Florianópolis, Santa Catarina, Brazil; 5 DDS, PhD. Department of Pathology, Health Sciences Center, Federal University of Santa Catarina, Florianópolis, Santa Catarina, Brazil

## Abstract

**Background:**

The glandular odontogenic cyst (GOC) is a rare aggressive odontogenic lesion, whose diagnosis can be challenging due to the overlap of microscopic findings with other lesions of the jaws. However, the main histopathological and immunohistochemical criteria for establishing the proper diagnosis have not yet been universally accepted. This study aimed to report a series of seven cases of Glandular Odontogenic Cyst, with emphasis on the comprehensive clinicopathological and immunohistochemical characterization.

**Material and Methods:**

Clinicopathologic data were gathered from medical records and histological slides obtained from paraffin-embedded samples of GOC. Additionally, the slides were subjected to immunohistochemical staining for cytokeratins (CK) 5, 7, 8/18, 19 and a cytokeratin pool (pan-CK), as well as for p63, α-SMA, and Ki67.

**Results:**

GOC occurred predominantly in females (71.42%), with a mean age of 48.28 ± 17.67 years, affecting the anterior region of the mandible (42.85%). Radiologically, the lesions were mostly unilocular (71.42%), showing buccal cortical bone expansion (85,71%). The main histological features included intraepithelial duct-like and crypt formations, apocrine metaplasia, and epithelial thickenings of the cystic lining. All lesions were strongly positive for pan-CK, CK5, and CK19, and moderately positive for p63. Ki67 was expressed in less than 5% of epithelial cells.

**Conclusions:**

Although many histopathological features of GOC have been reported, the presence of duct-like structures and crypts, and focal epithelial thickenings are likely the main diagnostic criteria of this lesion. Furthermore, the correlation of these features with immunohistochemical positivity for pan-CK, CK5, Ck19, and p63 may help establish the proper differential diagnosis of this odontogenic cyst.

** Key words:**Odontogenic cysts, differential diagnosis, oral pathology, immunohistochemistry.

## Introduction

Glandular odontogenic cyst (GOC) is a rare entity of the maxillary bones, constituting approximately 0.4% of all odontogenic cysts (1). Derived from the odontogenic epithelium, it has been added to the World Health Organization’s classification of odontogenic tumors as a developmental cyst exhibiting epithelial features simulating salivary gland or glandular differentiation, a classification that persisted until the most recent edition (1).

GOC exhibits a slight male prevalence (ratio of 1.15:1) and predominantly affects the anterior mandible in individuals in their fifth and sixth decades of life (2). Initially asymptomatic, larger cysts can become painful (2). Radiographically, GOCs typically appear unilocular with uniform radiolucency and well-demarcated borders, often leading to cortical bone expansion (2). The final diagnosis is based on a combination of clinical, imaging, and histopathological findings. Histologically, GOCs are characterized by stratified squamous epithelium with intraepithelial crypts, goblet cells, hobnail cells, epithelial plaques, microcysts or duct-like structures, and mucous or clear cells (3).

Accurate recognition and diagnosis of GOC is essential due to its potential for locally aggressive behaviour and a propensity for recurrence (4,5). Nevertheless, despite the diagnosis of GOC relying on the correlation of clinicopathological findings, there are some clinical and histopathological overlaps between GOC and other odontogenic cysts (e.g., dentigerous cyst, lateral periodontal cyst, botryoid odontogenic cyst), as well as non-odontogenic cysts (e.g., surgical ciliated cysts) and tumors, such as the cystic variant of low-grade central mucoepidermoid carcinoma, which require specific therapeutic approaches, and cystic extrafollicular adenomatoid odontogenic tumor (6-8).

The purpose of this study is to outline the clinical, radiographic, and histopathologic features and immunohistochemical profile of seven recently identified cases of GOC, including some instances with uncommon histopathological features.

## Material and Methods

A total of seven cases diagnosed histopathologically as GOC were retrieved from the files of the Laboratory of Oral Pathology at the Health Sciences Center, Federal University of Santa Catarina (Florianópolis, Santa Catarina, Brazil). The local Research Ethics Committee approved this study protocol (approval number: 42095715.1.0000.0121) and additional written informed consent was obtained. Two examiners independently reviewed all hematoxylin/eosin-stained histological slides under a light microscope. The diagnosis of GOC was confirmed based on the unequivocal identification of the major histopathological criteria reported by Kaplan *et al*. (3).: i) non-keratinized squamous epithelial lining with a flat interface; ii) presence of “spherules”/knobs or “whorls” or focal luminal proliferation; iii) epithelial lining exhibiting surface cuboidal eosinophilic cells or “hob-nail” cells; iv) mucous/goblet cells with intraepithelial mucous pools with or without crypts lined by mucous producing cells; and v) intraepithelial glandular microcystic or duct-like (pseudo-glandular) structures. Histochemistry for periodic acid-Schiff (PAS) was also performed to confirm the presence of mucous/goblet cells. Subsequently, clinical (age, sex, race, and site of lesion) and radiographic features (image location, cortical expansion, cortical perforation, involvement of dental crowns, tooth displacement, and root resorption) of each case were included and analysed.

For histological analysis, both the major diagnostic criteria and other microscopic features previously reported in cases of GOC, such as papillary proliferation, ciliated cells, stellate reticulum of the enamel organ differentiation, multicystic or multiluminal architecture, clear or vacuolated cells in basal or spinous layer, and subepithelial hyalinization, were classified as absent (-), mild (+), moderate (++), or abundant (+++).

For immunohistochemical analysis, deparaffinized 3 μm thick histological slides underwent endogenous peroxidase activity blockade with 3% hydrogen peroxide and methyl alcohol (10 min in a dark room). Subsequent antigen retrieval was performed by moist heat under pressure in 10 mM citrate buffer/pH 6.0 solution. The histological sections were incubated with primary antibodies ( Table 1). The secondary antibody (Streptavidin Biotin Complex, catalog number SA1022) was incubated at 37 ºC for 30 min. The reaction was revealed by incubating the histological slides with diaminobenzidine (DAB, Ventana Medical Systems, Tucson, AZ, USA) in a darkroom for 3 min. Counterstaining was performed with Meyer’s hematoxylin. The clinicopathological and immunohistochemical analyses were carried out independently by two observers who were blinded to the data.

## Results

 Table 2 summarizes the clinicopathologic data of the seven cases of GOC. The majority of the patients were women (71.42%), biracial (57.14%), with ages ranging from 24 to 78 years old (mean of 48.28 ± 17.67 years). None of the patients reported experiencing painful symptoms. GOC was more frequently located in the anterior region of the jaws, predominantly affecting the mandible (42.85%).

**Figure 1:**
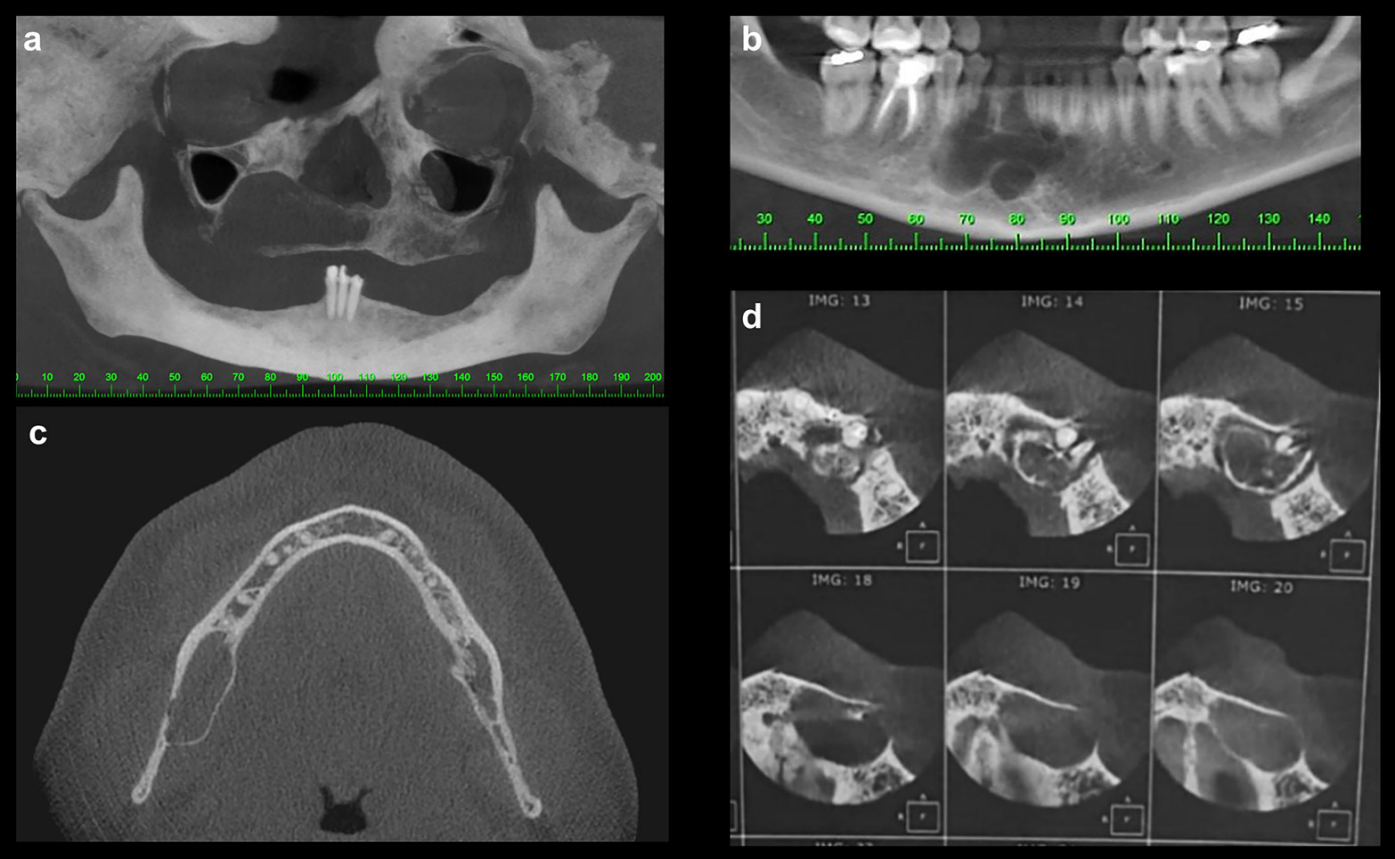
Main imaging features of glandular odontogenic cysts. (a) Well-delimited unilocular lesion in the posterior region of the maxilla. (b) Multilocular lesion in the anterior region of the mandible. (c) Unilocular lesion causing thinning of cortical bones and bulging of the lingual cortical. (d) Lesion located in the anterior region of the maxilla promoting buccal cortical expansion and perforation.

Analysis of the imaging characteristics of the lesions revealed a considerable variety of findings. Lesions with unilocular radiographic appearance (71.24%) were more common than multilocular ones. Expansion of the buccal bone cortex was observed in six cases (85.71%), while in two cases, the lingual/palatal cortex was bulging. Thinning of cortical bones was seen in all cases analysed, but perforation was observed in only two of them (one in the maxilla and the other in the mandible). In one case, the lesion presented atypical aggressive behavior, reaching large proportions, and extending from the region of the left first molar to the right second premolar, thus crossing the mandibular midline (Fig. 1). On an imaging basis, the mean size of lesions was 48.2 ± 24.7 mm.

**Figure 2:**
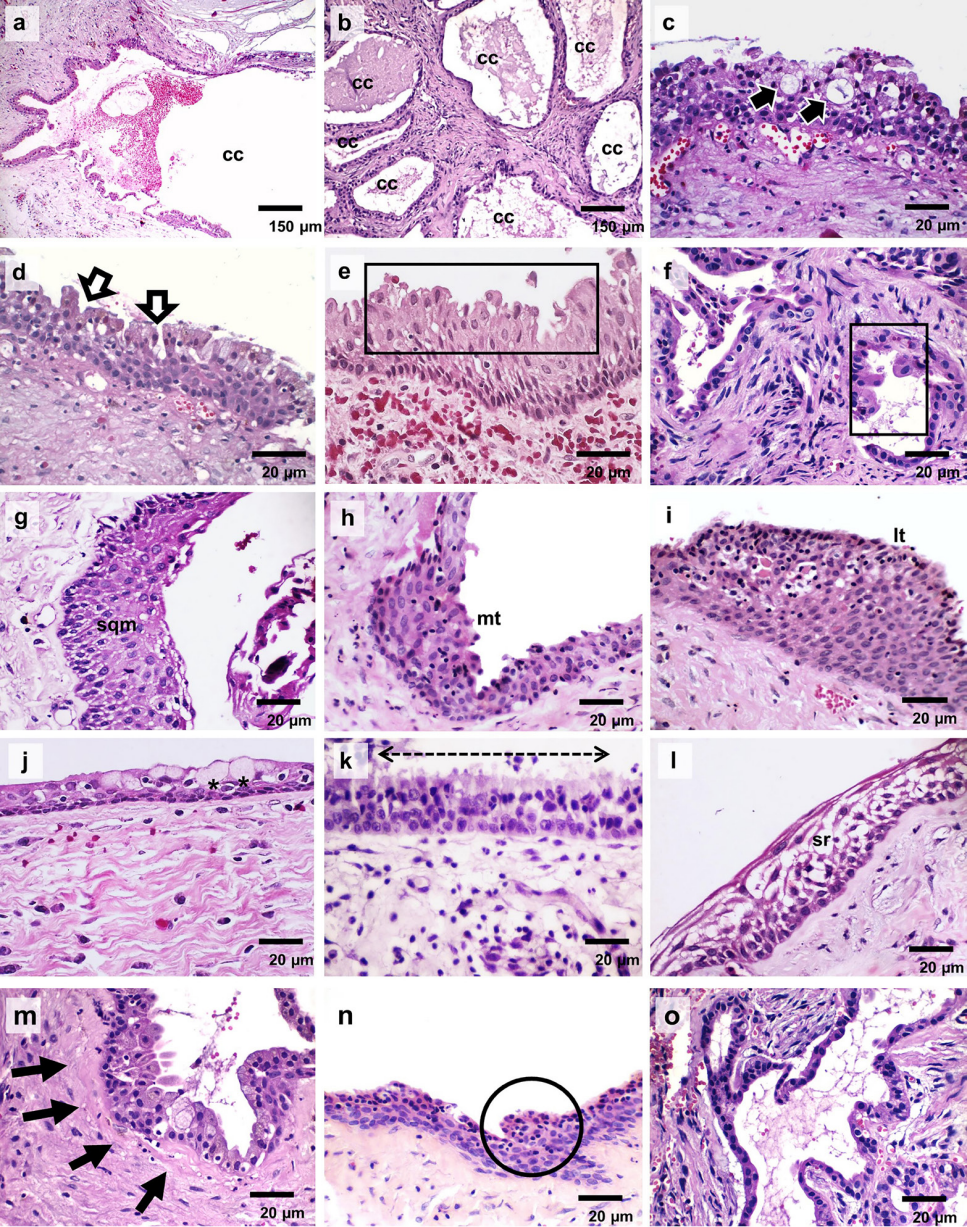
Histological sections show the observed histopathological features in the current series of glandular odontogenic cysts. Cystic lesions formed by (a) single and (b) a multicystic lesion formed by multiple small cavities resembling a neoplastic cystic lesion (A-B, 100x). (c) Pseudoductal structures, (d) and epithelial crypts, (e/f) apocrine metaplasia, (g) squamous metaplasia, and both (h) mural and (i) luminal plaque-like epithelial thickenings were histopathological findings observed in all the cysts (c-i, 400x). (j) Mucous differentiation, (k) ciliated pseudostratified columnar epithelium, (l) epithelial changes resembling the stellate reticulum of the enamel organ, (m) subepithelial hyalinization, (n) epithelial whorls and (o) papillary projections (“tufting”) were less frequently observed histopathological findings (j-n, 400 x; o, 100x). Subtitles: cc – cystic cavity; thick black arrows – duct-like structures; thick white arrows – epithelial crypts; rectangle – apocrine metaplasia; sqm – squamous metaplasia; mt – mural epithelial thickening; lt – luminal epithelial thickening; (*) mucous differentiation; dashed line – ciliated epithelium; sr – degenerative changes resembling the stellate reticulum of the enamel organ; thin black arrows – subepithelial hyalinization; circle – epithelial whorls.

Histopathological analysis of the lesions is summarized in  Table 3. Most cases consisted of a single pathological cavity (51.14%), with two or more cystic cavities being observed in three cases. Case six was the only one to present multiple small cavities lined by epithelium, without the formation of a larger cavity, resembling a neoplastic lesion. Pseudoducts, epithelial crypts, apocrine and squamous metaplasia, as well as both luminal and mural plaque-like epithelial thickenings, were histopathological features observed in all lesions analysed, although with varying intensity (Fig. 2). Mucous cells were observed in three cases (42.85%), whereas ciliated cells and areas of epithelial changes resembling the stellate reticulum of the enamel organ were both seen in only one case (14.28%). The cysts’ capsule was composed of fibrous connective tissue exhibiting mild chronic inflammation in three cases (42.85%) and moderate in one (14.28%). Some areas of oedematous changes were found in two cases (28.57%), whereas diffuse oedema of moderate intensity and cholesterol clefts associated with foreign body reaction were present in one case (14.28%). Focal haemorrhage areas were observed in all cases, whereas dystrophic calcification was identified in only one case.

**Figure 3:**
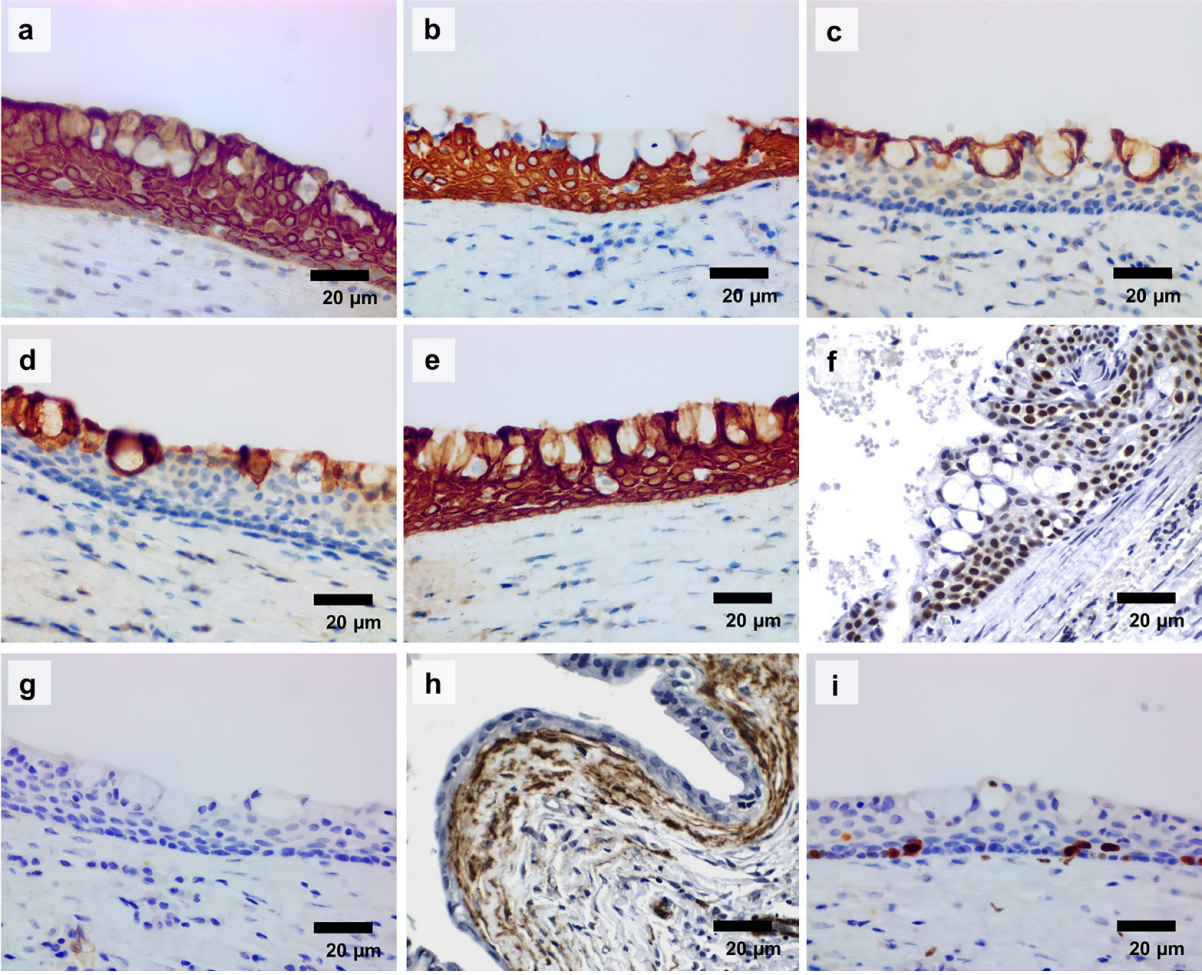
Immunohistochemical profile of glandular odontogenic cysts. (a) Pan-cytokeratin (AE1/AE3) diffusely expressed in the cytoplasm of all epithelial cells. (b) CK5 expressed in squamous and basal cells. (c) CK7 and (d) CK8/18 expressed in mucous/goblet cells. (e) CK19 expressed in all epithelial cells of the cystic lining. (f) Nuclear immunoexpression of p63 seen in squamous and basal cells. (g) Case showing negativity for α-SMA. (h) Case showing strong immunoreactivity for α-SMA in the subepithelial connective tissue of the fibrous capsule. (i) Weak immunoexpression of Ki67, limited to basal and parabasal cells of the cystic epithelium (SABC, 400 x).

The immunohistochemical expression profile of the GOC is presented in Figure 3 and  Table 4. In this series, all the cases (100.0%) showed cytoplasmic positivity for AE1/AE3 and CK19 throughout the cystic epithelium, whereas CK5 was strongly positive in squamous and basal cells. Cytoplasmic expression of CK7 and CK8/18 was observed in three cases (42.85%), but it was weak to moderate and restricted to epithelial cells exhibiting mucous differentiation and lining epithelial crypts and pseudoductal structures. Nuclear positivity for p63 ranged from moderate to intense in 100.0% of the cases, in squamous and basal cells, whereas for ki67 the immunopositivity was weak (<5%) in basal and parabasal epithelial cells. Only case six showed positivity for α-SMA, but only in spindle cells of fibrous connective tissue arranged in a narrow subepithelial band.

## Discussion

The current paper describes a series of seven cases of GOC, a rare cystic entity of odontogenic origin and aggressive behaviour, comprising slightly less than 2% of odontogenic cysts (3). GOC displays some pathological features of salivary gland tumors, such as mucous cell differentiation and the formation of duct-like epithelial structures in the cyst lining (3). Due to its aggressive behaviour and histopathological presentation, the differential diagnosis between GOC and low-grade mucoepidermoid carcinoma, the most common malignant salivary gland tumor, can be sometimes quite laborious (9,10). Therefore, the precise characterization of this odontogenic cyst is crucial for a proper diagnosis and prompt treatment.

In this case series, we observed a higher prevalence in females, consistent with the findings of Martins-Chavez *et al*. (11), whereas in a larger series of 46 cases previously reported by Fowler *et al*. (9) the distribution of cases between men and women was fairly homogeneous (a ratio of approximately 1.04:1.0). On the other hand, a systematic review comprising 169 cases of GOC, along with a smaller series of five cases, reported a predilection for males(2). Despite the wide variation in the age range, the average age of the patients was close to the end of the fifth decade of life, which agrees with other studies (2,11,12). The most common clinical presentation of GOC was painless swelling in the anterior region of the mandible, a finding supported by previous reports in most studies (2,9,11).

Regarding the imaging features the most frequently observed findings in the current series were well-delimited unilocular radiolucency promoting buccal expansion and thinning of the mandibular cortical bone. A similar imaging spectrum has been described by Nel *et al*. (12)based on clinical and radiological analysis of 92 cases. However, unlike these last authors who observed loss of cortical bone integrity in 71% of cases, this finding — associated with aggressive clinical behaviour — was only evidenced in two cases in the current study (28.57%). Moreover, although GOCs involving unerupted teeth, mimicking a dentigerous relationship(7), and odontomas (6) have been well-documented, none of these associations were observed in the current series.

Although the microscopic features of GOC have been extensively documented, including the most recent World Health Organization classification (1,13), the correct diagnosis of this cyst may be challenging. It can be confused with lateral periodontal cyst, botryoid odontogenic cyst, radicular and residual cysts with mucous metaplasia, and low-grade mucoepidermoid carcinoma (14).

According to Kaplan *et al*. (3), the major histopathological criteria to establish the diagnosis of glandular odontogenic cyst are non-keratinized squamous epithelial lining with a flat interface, presence of “spherules”/knobs or “whorls” or focal luminal proliferation; epithelial lining exhibits surface cuboidal eosinophilic cells or “hob-nail” cells; mucous/goblet cells with intraepithelial mucous pools with or without crypts lined by mucous producing cells; and intraepithelial glandular microcystic or duct-like (pseudo-glandular) structures. All cases reported in the current paper met all these criteria.

Nevertheless, some histopathological findings considered as “minor criteria” (3) were also observed in variable proportions in some cases reported in this study, such as papillary proliferation, ciliated cells, multicystic or multiluminal architecture and, vacuolated cells in basal and spinous layer (resembling the stellate reticulum of the enamel organ). Although these minor criteria may help facilitate histopathological recognition of GOC, they are not essential for diagnosis.

The histopathological changes identified in the cystic capsule, such as inflammation, oedema, and haemorrhage, were uncommon and represented only incidental findings. Those can be found in any jaw cyst, particularly when they are secondarily infected. Therefore, they have no significance for the diagnosis of GOC. Cholesterol clefts were also observed in the current case, but they were associated with inflammation of the connective tissue. Interestingly, the aforementioned findings have been recently described in non-inflamed cases of GOC (15) but its significance has not been clarified yet.

The assessment of the immunohistochemical profile is a useful tool for establishing the differential diagnosis of odontogenic cysts, particularly the expression of cytokeratins (16,17). In the current study, we observed intense and diffuse immunohistochemical positivity for pan-cytokeratin (AE1/AE3), CK5, and CK19 throughout the cystic epithelium of GOCs. The wide spectrum CK (AE1/AE3) is frequently used to determine the cell epithelial nature, but it is not appropriated as a marker of epithelial origin (e.g., odontogenic, glandular, etc.). On the other hand, although positive immunoexpression of CKs 5, 7, 13, 14, and 19 have been reported in different embryological structures and stages of odontogenesis, CK5, 14 and CK19 seem to be the major biomarkers, not only of odontogenic differentiation (18) but also of development cysts derived from the odontogenic epithelium (19). Therefore, our findings confirm the odontogenic origin of the cysts.

We also found focal immunoexpression of CK7, and CK8/18 in cases of GOC, particularly those with mucous differentiation in the cystic epithelial lining. The immunoexpression of these cytokeratins is controversial, with some studies showing focal positivity (10,20), and others being negative (17). CK7 is expressed during salivary gland development and retained in adult salivary glands (21). Additionally, CK8/18 is a mucous epithelial keratin expressed both in mucous acini and in simple non-stratified and secretory epithelium, such as salivary gland ducts. Thus, CK7, CK8, and, CK18 are considered the most useful markers of salivary gland differentiation, including benign (22) and malignant tumors (23,24). Hence, the pattern of CK7 and CK8/18 expression found in the current study suggests that the positive cell area likely results of mucous metaplasia. Furthermore, the lack of positivity in luminal cells of the epithelial duct-like structures, in opposition to their positivity for CK19, is suggestive of odontogenic rather than glandular origin, confirming that they do not represent true ducts. Analysis of the CK7, CK8/18 and, CK19 expression profiles can also be useful adjunctive tools in distinguishing GOC from low-grade mucoepidermoid carcinoma, the most challenging differential diagnosis of this odontogenic cyst (10).

A predominantly moderate expression of p63 was observed in basal, parabasal, and squamous cells in GOC. The immunohistochemical expression of p63 has been proposed not only to play a major role in the maintenance of epithelial stem cells and their terminal differentiation (ΔN P63 isoforms, lacking N-terminal transactivation domain) but also to be involved in the proliferative activity of the odontogenic epithelium (TAP63 isoforms, lacking the transactivation domain) in cysts and tumors (25). In fact, p63 overexpression has been associated with increased proliferation and is regarded as a potential prognostic marker in odontogenic lesions with more aggressive and invasive phenotypes (26). However, as in the current study, ki67 immunoexpression, a well-recognized biomarker of cell proliferation, was low and heterogeneous, and not related to p63 expression. Therefore, no direct relationship with proliferative potential or clinical aggressiveness of the cysts could be established.

Interestingly, p63 is also considered a marker of myoepithelial differentiation, including in salivary gland tumors (27). However, the most reliable definition of myoepithelial phenotype indicative of the histogenesis of salivary gland origin is based on a simultaneous positiveness for p63 and α-SMA (alpha-smooth muscle actin) (28). Thus, the lack of positivity for α-SMA in the epithelial cells of the cystic lining rules out any possibility of myoepithelial differentiation in these lesions. The identification of α-SMA-positive cells, interpreted as myofibroblasts, in the fibrous capsule of odontogenic cysts has been previously reported (29). Increased counts of SMA-positive cells in the fibrous capsule of odontogenic keratocyst and ameloblastoma, in comparison with dentigerous cysts, suggest a possible association of myofibroblasts with the epithelial cells. This association may contribute to changes in the stromal microenvironment, favouring the growth and progression of the lesion.(30). However, in the current study, only one case of GOC demonstrated significant positivity for SMA in the cystic capsule. Interestingly, this positivity was observed not in the largest lesion or with the most aggressive clinical behaviour, but in the one with many cystic spaces, whose histological appearance was closest to a multicystic neoplastic lesion. Therefore, further studies are necessary to better characterize the role of myofibroblasts in the pathogenesis and clinical behaviour of GOC.

## Conclusions

GOC is a rare odontogenic cyst, and its variety of clinical manifestations and imaging aspects can make its diagnosis challenging. Given the major and minor histological criteria established for GOC, the presence of intraepithelial formations similar to microcystic ducts, epithelial crypts, and focal epithelial thickenings represent the main histopathological features. However, due to the similarity of GOC with low-grade multicystic mucoepidermoid carcinoma, evaluating the immunohistochemical profile of the lesion including pan-CK, CK5, CK19, and p63 is necessary to distinguish these entities. Thus, the correlation between the microscopic, imaging, and immunohistochemical findings is essential to establish an assertive diagnosis and determine the adequate management of the lesion.

## Figures and Tables

**Table 1:** Antibodies used in the immunohistochemical analysis of glandular odontogenic cysts.

Antigen	Label	Clone	Dilution	Incubation	Control
Anti Cytokeratin	Dako	AE1/AE3	1:1200	Overnight	Squamous epithelium of the oral mucosa
Anti CK5/6	Dako	D5/16 B4	1:200	Overnight	Mesothelioma
Anti CK7	Dako	OV-TL-12/30	1:100	Overnight	Salivary glands
Anti CK8/18	Dako	EP17/EP30	1:50	2 h	Liver
Anti CK19	Dako	RCK108	1:100	Overnight	Odontogenic keratocyst
Anti p63	Neomarkers	4A4	1:100	Overnight	Salivary glands
Anti Ki67	Dako	MIB-1	1:50	2 h	Oral hyperplastic lymph node
α-SMA	Dako	1A4	1:600	Overnight	Oral myofibroma

**Table 2:** Clinical and imaging features of the seven cases of glandular odontogenic cysts.

Clinical / Imaging features	Case 01	Case 02	Case 03	Case 04	Case 05	Case 06	Case 07
Anatomic site	Mandible	Mandible	Mandible	Mandible	Maxilla	Mandible	Maxilla
	Posterior	Anterior	Anterior	Anterior	Anterior	Posterior	Anterior
Age (years)	49	78	34	31	48	50	46
Sex	Male	Female	Female	Female	Male	Female	Female
Race	Biracial	Black	Biracial	White	Biracial	Biracial	Black
Imaging characteristic	Unilocular	Multilocular	Multilocular	Unilocular	Unilocular	Unilocular	Unilocular
Well-delimited edges	Yes	Yes	No	Yes	Yes	Yes	Yes
Buccal cortical expansion	Yes	Yes	Yes	Yes	Yes	No	Yes
Lingual/palatal cortical expansion	No	No	Yes	No	No	No	Yes
Cortical thinning	Yes	Yes	Yes	Yes	Yes	Yes	Yes
Cortical perforation	No	No	Yes	No	No	No	Yes

**Table 3:** Clinical and imaging features of the seven cases of glandular odontogenic cysts.

Histopathological features	Case 01	Case 02	Case 03	Case 04	Case 05	Case 06	Case 07
Epithelial lining							
Cystic cavity	Single	Multiple ^a^	Multiple ^a^	Single	Single	Multiple ^a^	Single
Pseudoducts	++	+++	++	++	++	+++	++
Epithelial crypts	+	+	+	+	++	+++	+
Apocrine metaplasia	++	+	+	+	+	+++	++
Mucous/goblet cells	-	-	-	-	+++	++	+++
Ciliated cells	-	-	-	-	+	-	-
Epithelial thickenings	++	+	++	+	++	+++	+
Epithelial whorls	+	-	+	-	+	-	-
Tufting (papillary projections)	-	-	+	+	-	++	
Stellate reticulum of the enamel organ Degenerative changes	-	+	++	-	-	-	-
Fibrous capsule	Case 01	Case 02	Case 03	Case 04	Case 05	Case 06	Case 07
Satellite microcysts	+	-	+	-	-	+++	-
Inflammation	+	+	-	-	++	+	-
Oedema		+^b^			+++	+ ^b^	
Cholesterol clefts	-	-	-	-	+	-	-
Haemorrhage	+	+	++	+	++	+	+
Dystrophic calcification	-	+	-	+ ^b^	-	-	-

a Lesions with two or more cystic cavities without apparent connection. b Limited to a few focal areas. Absent (-), mild (+), moderate (++), or abundant (+++)

**Table 4:** Assessment of the immunohistochemical profile of the seven cases of glandular odontogenic cysts.

Antibody	Immunohistochemical pattern	Case 01	Case 02	Case 03	Case 04	Case 05	Case 06	Case 07
AE1/AE3	Cytoplasm of all epithelial cells	+++	+++	+++	+++	+++	+++	+++
CK5	Cytoplasm of squamous cells	++	++	++	++	++	++	++
CK7	Cytoplasm of mucous/goblet cells	-	-	-	-	+	+	++
CK8/18	Cytoplasm of mucous/goblet cells	-	-	-	-	+	+	++
CK19	Cytoplasm of all epithelial cells	+++	+++	+++	+++	+++	+++	+++
p63	Nuclei of squamous cells	++	+	++	++	+	++	++
Ki67	Nuclei of basal and parabasal cells (<5%)	+	+	+	+	+	+	+
α-SMA	Cytoplasm of spindle cells of the cystic fibrous capsule (subepithelial pattern)	-	-	-	-	-	++	-

Absent (-), mild (+), moderate (++), or abundant (+++)

## Data Availability

The datasets used and/or analyzed during the current study are available from the corresponding author.
